# 2413: Immune stress biomarkers correlate to violence and internalization of violence in African American young adults

**DOI:** 10.1017/cts.2017.74

**Published:** 2018-05-10

**Authors:** Latifa Jackson, Max Shestov, Forough Saadatmand, Joseph Wright

**Affiliations:** Georgetown - Howard Universities, Washington, DC, USA

## Abstract

OBJECTIVES/SPECIFIC AIMS: Allostatic load, the chronic stress-induced wear and tear on the body, has a cumulative deleterious effect in individuals over their lifetime. Recent studies have suggested that socio-economic status, psychological determinants, and biomedical health cumulatively contribute to allostatic load in young adults. Although these finding individually suggest that African American children may be particularly susceptible to the effects of allostatic loading due to racially-based discrimination and economic instability, few studies have shown the effect of exposure to violence on the allostatic load carried by young African Americans. METHODS/STUDY POPULATION: The Biological and Social Correlates of Drug Use in African American Emerging Adults (BADU) data set is composed of young African Americans (n=557 individuals) living in the Washington, DC area, collected from 2010 to 2012. Study participants were sought equally between males and females (n=283, n=274, respectively). This data set provides a rich source of information on the behavioral, mental, and physical health of African American young adults (18–25 year olds) living in the Washington, DC area. Analysis of 6 biomedical markers were measured in BADU study participants: C-reactive protein, cortisol, Epstein-Barr virus IgG, IgE, IgA, and IgM, known to be markers of immune stress and allostatic load. Naive Bayes was used to identify participant responses that were correlated to elevated stress biomarker levels. RESULTS/ANTICIPATED RESULTS: Violence was most closely correlated to elevated EBVVCA IgM and IgE levels. Elevated IgE levels correlated to increased experience of familial violence and sexual abuse; familial drug abuse and depression; violence and community violence. Cortisol is positively correlated to reported emotional state (*R*=0.072) and perceived individual discrimination (*R*=0.059). DISCUSSION/SIGNIFICANCE OF IMPACT: Allostatic load appears to be high in individuals who self-report exposure to violence. Both perceived mental health and violence were correlated to elevated stress biomarkers. When Epstein-Barr virus viral capsid antigen IgM was compared with violence features characterized in the data set, we found that internalization of environmental stressors were most strongly correlated to elevated allostatic load markers. This work suggests that internalization of experienced violence may be as important as the actual violence experience.

